# Inhaled bispecific single-domain antibody BM219 for mild-to-moderate COVID-19: a double-blind, randomized, placebo-controlled phase 2 trial

**DOI:** 10.1038/s41421-025-00813-0

**Published:** 2025-07-17

**Authors:** Yanling Wu, Yuan Li, Ping Zhang, Siwei Guo, Fang Yuan, Vivian Liu, Ting Yu, Feng Lin, Nan Yang, Chao Tu, Hongzhou Lu, Tianlei Ying, Xin Li

**Affiliations:** 1 Key Laboratory of Medical Molecular Virology (MOE/NHC/CAMS) and Shanghai Institute of Infectious Disease and Biosecurity, School of Basic Medical Sciences, Department of Pulmonary and Critical Care Medicine, Department of Liver Surgery and Transplantation, Zhongshan Hospital, Fudan University, Shanghai, China; 2 Department of Pharmacy, The Third Hospital of Changsha, Changsha, Hunan China; 3 Biomissile (Anji) Pharmaceuticals Co., Ltd, Anji, Zhejiang China; 4 Department of Infectious Diseases, Hainan General Hospital, Hainan Affiliated Hospital of Hainan Medical University, Haikou, Hainan China; 5 The Third Hospital of Hebei Medical University, Shijiazhuang, Hebei China; 6 National Clinical Research Center for Infectious Diseases, The Third People’s Hospital of Shenzhen and The Second Affiliated Hospital of Southern University of Science and Technology, Shenzhen, Guangdong China; 7 Shanghai Engineering Research Center for Synthetic Immunology and Shanghai Key Laboratory of Lung Inflammation and Injury, Shanghai, China

**Keywords:** Immunology, Biological techniques

Dear Editor,

Since the outbreak of coronavirus disease 2019 (COVID-19) in late 2019, severe acute respiratory syndrome coronavirus 2 (SARS-CoV-2) has undergone rapid evolution, resulting in substantial changes in transmissibility, pathogenicity, and immune escape potential. This ongoing viral diversification has largely undermined the protective efficacy of existing vaccines and neutralizing antibodies, posing major challenges to the clinical management of COVID-19^1,2^. Several monoclonal antibodies previously granted emergency use authorization by the U.S. Food and Drug Administration for the treatment of mild-to-moderate COVID-19 have now been restricted or withdrawn due to their markedly reduced activity against emerging variants, particularly those from the Omicron lineage. Among these, the Omicron subvariant JN.1 has attracted global attention and was designated a variant of interest by the World Health Organization in December 2023, shortly after its emergence^3^. Owing to its extensive antigenic divergence, the JN.1 variant demonstrates broad resistance to class 1, 2, and 3 receptor-binding domain (RBD)-targeting antibodies^4^. Its enhanced immune evasion has contributed to its rapid rise as a predominant circulating strain worldwide, highlighting the urgent need for next-generation antiviral strategies capable of retaining efficacy against highly evolved SARS-CoV-2 variants.

SARS-CoV-2 predominantly infects the respiratory tract, and COVID-19 severity is largely driven by respiratory rather than systemic pathology^5^. However, all clinically approved neutralizing antibodies to date are administered systemically, with less than 0.2% reaching the lungs, necessitating high doses (up to gram levels) to achieve therapeutic efficacy^6,7^. This approach increases both cost and risk of systemic toxicity. Inhalation offers a more efficient alternative, enabling direct delivery of antibodies to the site of infection, improved pulmonary bioavailability, and reduced systemic exposure^8^. Despite its potential, no inhaled antibody product has yet been approved for clinical use, highlighting the need for further translational development.

To address these challenges, we developed BM219 (also known as bn03), a fully human, bispecific single-domain antibody that simultaneously targets two distinct, non-overlapping epitopes within the RBD of the SARS-CoV-2 spike protein^9,10^. One of these epitopes is highly conserved and structurally buried within the trimeric interface, conferring broad neutralizing activity across variants. BM219 has shown potent in vitro neutralization against all major SARS-CoV-2 strains, from the ancestral lineage to circulating Omicron subvariants (Supplementary Fig. S1)^11^. Its small molecular size and high thermal stability make it particularly suitable for aerosol administration via nebulization. Here, we report results from a double-blind, randomized, placebo-controlled phase 2 trial (ChiCTR2400094206, CTR20233768) evaluating the safety and efficacy of inhaled BM219 in adults with mild-to-moderate COVID-19.

A total of 120 patients were screened over a six-month period across six centers starting between November 24, 2023 and April 25, 2024. Of these, 36 were excluded for not meeting the inclusion criteria. The remaining 84 patients from four centers (Supplementary Table S1), all confirmed SARS-CoV-2 positive by nucleic acid testing, were enrolled. Participants were randomized to receive either placebo (*n* = 24) or one of three BM219 regimens: 60 mg twice daily (BID), 120 mg once daily (QD), or 120 mg BID (*n* = 20 per group; Fig. 1a). All treatments were initiated within 5 days of symptom onset. The mean age of enrolled patients was 33.4 ± 8.7 years; 45 (53.6%) were male and 39 (46.4%) female. All participants had experienced at least one prior episode of COVID-19 and had received various COVID-19 vaccines. Viral sequencing identified the infecting strain as JN.1 in 59.5% (50/84), EG.5 in 4.8% (4/84), wild-type or other variants in 23.8% (20/84), and unknown in 11.9% (10/84) of cases. On day 1, the mean Ct values were 23.6 for the N gene and 24.4 for the ORF gene (Supplementary Table S2).

**Fig. 1:**
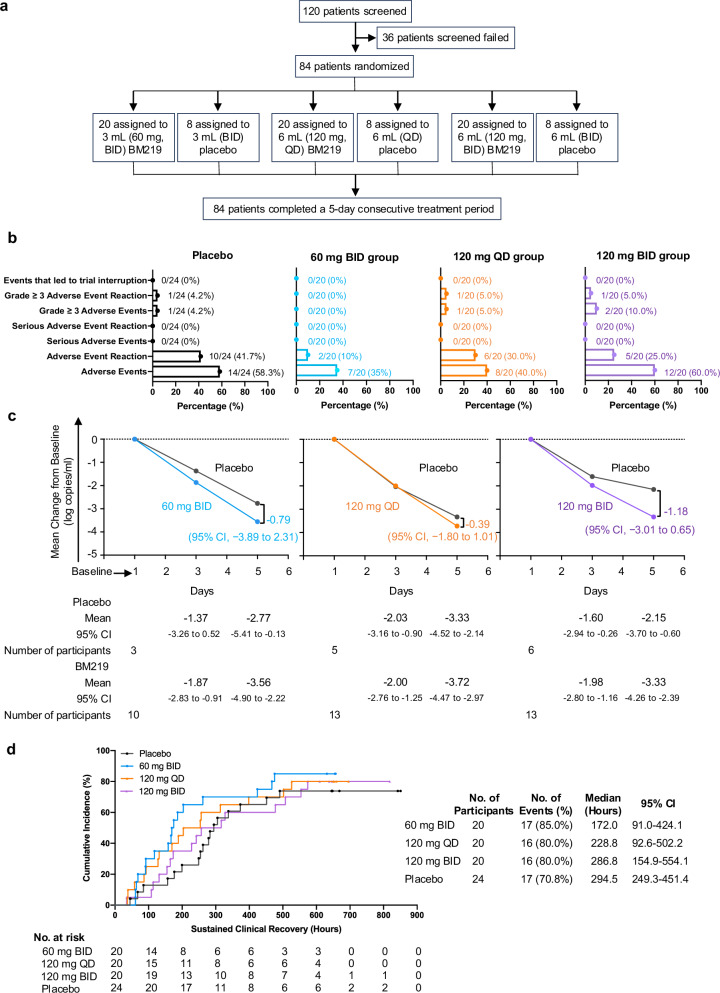
Safety and efficacy of inhaled bispecific single-domain antibody BM219 for mild-to-moderate COVID-19. **a** Trial profile. Patients with mild-to-moderate COVID-19 were recruited to assess the efficacy of inhaled BM219. A total of 84 participants were randomized into three cohorts to receive BM219 at 60 mg twice daily, 120 mg once daily, 120 mg twice daily. Within each cohort, participants were further assigned to receive either BM219 or a matching placebo at a 5:2 ratio over a 5-day treatment period. **b** Adverse events occurring from the first dose through 28 days. Bar graphs present the percentage of participants reporting adverse events (AEs), serious AEs (SAEs), grade ≥ 3 AEs, and events leading to trial interruption, stratified by treatment group (60 mg BID, 120 mg QD, 120 mg BID, and placebo). Denominators indicate group sample sizes (*n* = 20 or *n* = 24). AE categories are defined in protocol. **c** Change in viral load from baseline through day 5 in the JN.1-infected patients over time. The viral load was quantified by RT-PCR of nasopharyngeal swabs, with Day 1 (pre-first dose) defined as baseline. Shown are the mean absolute changes in viral load (log10 copies/mL) from baseline through Day 5 for each treatment group (60 mg BID, 120 mg QD, 120 mg BID) and the corresponding placebo group among JN.1-infected participants. **d** Time to sustained clinical recovery in the full analysis population. Final analysis was performed using the Kaplan–Meier method. Shown is the cumulative incidence of time to sustained clinical recovery (hours) for placebo, 60 mg BID, 120 mg QD, and 120 mg BID groups. Sustained clinical recovery was defined as the alleviation of all COVID-19-related symptoms to a total score of 0 or 1 (sum of 11 symptoms, each scored 0–3; total range 0–33) maintained for at least 2 consecutive days. Data include number of participants at risk at each timepoint, event rates (*n*, %), median times with 95% confidence intervals (95% CI), and comparative treatment trajectories.

The overall incidence of adverse events (AEs) was 45.0% (27/60) in the BM219 treatment groups, comparable to 58.3% (14/24) in the placebo group (*P* = 0.3) (Fig. 1b). Most AEs were grade 1 or 2. Common AEs (≥ 5% incidence in any group) included elevated blood triglycerides (3 (12.5%) patients in the placebo group vs 1 (5.0%) in the 120 mg QD group) and hypertriglyceridemia (1 (5.0%) in the 60 mg BID group vs 2 (10.0%) in the 120 mg QD group; Supplementary Table S3), which may be related to individual lifestyle or underlying metabolic condition. The incidence of grade ≥ 3 AEs was 0% in the 60 mg BID group, 5.0% (1/20) in both the 120 mg QD and 120 mg BID groups, and 4.2% (1/24) in the placebo group (Fig. 1b). No serious AEs were reported in any group, and no trial interruptions or participant withdrawals occurred due to AEs. Collectively, BM219 was well tolerated, with no safety concerns related to the inhalation procedure or the compound. Most events were mild to moderate in severity which is notably lower than those typically observed with systemically delivered monoclonal antibodies or antivirals.

Next, we assessed the effect of BM219 on viral load, resolution of symptoms, and disease progression. At day 5, the mean change from baseline viral load compared to placebo was −0.08 log_10_ copies/mL (95% CI, −1.61 to 1.46; *P* = 0.92) in the 60 mg BID group, −0.29 log_10_ copies/mL (95% CI, −1.49 to 0.9; *P* = 0.61) in the 120 mg QD group, and 0.32 log_10_ copies/mL (95% CI, −1.59 to 2.22; *P* = 0.73) in the 120 mg BID group in the full analysis set (FAS) population (Supplementary Table S4). The modest changes observed across all groups were likely due to widespread pre-existing immunity, as all participants had at least one prior episode of COVID-19 and had received various vaccinations. This is consistent with previous studies showing that prior infection and immunization are associated with limited viral load reductions in clinical trials due to baseline seropositivity^7^. As the trial was conducted during a wave dominated by the immune-evasive Omicron JN.1 variant, which accounted for 59.5% (50/84) of enrolled patients, we performed a subgroup analysis in this population, given that its high level of immune escape compromises the efficacy of pre-existing neutralizing antibodies. In the JN.1 subgroup, viral load reductions were more pronounced. At day 5, the 120 mg BID group showed the largest reduction from baseline compared to placebo (−1.18 log_10_ copies/mL; 95% CI, −3.01 to 0.65; *P* = 0.19), followed by the 60 mg BID group (−0.79 log_10_ copies/mL; 95% CI, −3.89 to 2.31; *P* = 0.58) and the 120 mg QD group (−0.39 log_10_ copies/mL; 95% CI, −1.80 to 1.01*; P* = 0.56) (Fig. 1c). The pharmacokinetic analyses further demonstrated antibody concentrations in induced sputum at Day 5 were highest in the 120 mg BID group (~26,000 ng/mL), followed by the 60 mg BID group (~15,000 ng/mL), with the 120 mg QD group showing the lowest levels (~6000 ng/mL) (Supplementary Fig. S2). These findings are consistent with the observed antiviral efficacy and indicate that BM219, particularly at the 120 mg BID dose, achieved meaningful viral suppression in patients infected with JN.1, with efficacy greater than that for previously reported inhaled monoclonal antibody cocktails against earlier SARS-CoV-2 variants^12^.

Time to sustained clinical recovery and SARS-CoV-2 negativity was further evaluated in 49 (81.7%) of 60 patients receiving BM219 and 17 (70.8%) of 24 patients in the placebo group. Median time to sustained clinical recovery was shortest in the 60 mg BID group at 172.0 h (95% CI, 91.0 to 424.1; *P* = 0.4), compared to 294.5 h (95% CI, 249.3 to 451.4) in the placebo group, representing a reduction of over 5 days. The 120 mg QD (228.8 h; 95% CI, 92.6 to 502.2; *P* = 0.8) and 120 mg BID (286.8 h; 95% CI, 154.9 to 554.1; *P* = 1.0) groups also showed faster recovery, though to a less extent (Fig. 1d). Time to viral negativity was also reduced, with reduction range from 68.3 to 76.6 h in all BM219 groups relative to placebo (Supplementary Fig. S3). Clinical outcomes, as determined by alleviation of COVID-19-related symptoms, were improved in participants receiving BM219. Notably, the 60 mg BID group achieved the fastest symptom resolution, with performance comparable to or better than previously approved therapies, including REGEN-COV (14 days)^13^, bamlanivimab-based regimens (8 days)^14^, and oral antivirals such as nirmatrelvir-ritonavir, ensitrelvir, and simnotrelvir (7 days)^15^. Notably, although the 120 mg BID group demonstrated greater antiviral activity in terms of viral load reduction, this did not translate into faster symptom resolution compared to the 60 mg BID group, possibly due to multiple factors including host-related immune responses or disease variability. These findings suggest that the 60 mg BID regimen may offer a more favorable balance between anti-viral efficacy and clinical symptom resolution outcomes.

In summary, this trial provides the first clinical evidence that inhaled bispecific single-domain antibodies are safe, well-tolerated, and potentially effective against immune-evasive SARS-CoV-2 variants. BM219 improved clinical outcomes without increasing adverse events, with particularly encouraging results observed in JN.1-infected patients. A phase 3 trial is currently in preparation to further assess its efficacy and long-term benefits in a broader patient population. Considering the immune evasion associated with currently available antibodies, the broad-neutralizing BM219 represents a promising therapeutic option against circulating SARS-CoV-2 variants. This study also underscores the potential of inhaled single-domain antibodies as a novel modality for the treatment of COVID-19 and other respiratory viral infections.

## Supplementary information

Supplementary Information
