# Supplementary material for: Inhaled bispecific single-domain antibody BM219 for mild-to-moderate COVID-19: a double-blind, randomized, placebo-controlled phase 2 trial
Source: Cell Discov. 2025 Jul 17;11:64. doi: 10.1038/s41421-025-00813-0 (PMC12271308; doi:10.1038/s41421-025-00813-0)
Supplement: Supplementary file 1 — Supplementary Information [file 41421_2025_813_MOESM1_ESM.pdf]

Supplementary Materials

Inhaled bispecific single-domain antibody BM219 for mild-to-moderate COVID-19: a double-blind, randomized, placebo-controlled phase 2 trial

Yanling Wu, Yuan Li, Ping Zhang, Siwei Guo, Fang Yuan, Vivian Liu, Ting Yu, Feng Lin, Nan Yang, Chao Tu, Hongzhou Lu, Tianlei Ying, Xin Li

Methods and Materials

Trial design

This multicenter, double-blind, randomized, placebo-controlled phase 2 trial was conducted in patients with mild-to-moderate COVID-19 in China during a period from November 24, 2023 to April 25, 2024. 84 eligible patients with COVID-19 were enrolled within 4 centers in China (number of patients at each center shown in Supplementary Table S1). The trial was carried out in accordance with all applicable national and local regulatory requirements. The study protocol was approved by all participating sites (approval number: 2023-035 for Shenzhen Third People's Hospital; 2023EC-035 for The Third Hospital of Changsha; 2023-76-01 for Hainan General Hospital; and 2023-048-1 for The Third Hospital of Hebei Medical University). Written informed consent was obtained from all trial participants. Trial reporting conformed with the CONSORT guideline.

Participants

The adult patients (18 to 70 years of age) with a diagnosis of mild or moderate COVID-19, determined based on symptom scores (Supplementary Table S5) and quantitative RT-PCR analysis of oropharyngeal swabs, were randomized at a 5:2 ratio to receive BM219 at 60 mg BID, 120 mg QD, 120 mg BID, or matching placebo for 5 consecutive days. Additional inclusion criteria included: 1) cycle threshold (Ct) value <30 for the open reading frame 1ab (ORF1ab) or nucleocapsid (N-gene), and 2) symptom onset within 3 days. Key exclusion criteria included: 1) the use of other anti-viral drugs, 2) known allergy to antibody drugs, and 3) immunodeficiency subjects. For full details of the eligibility criteria are listed in the study protocols as shown below.

Randomization and masking

The study was done as a blinded study. Concealment was conducted using an interactive web service when a given patient was enrolled. All participants, investigators, the sponsor's study team, and laboratory staff were masked to group allocation before the final database lock at the conclusion of trial. Randomization was

conducted at a 5:2 ratio. Randomization sequence was generated by a statistician not involved in this trial otherwise using the SAS software (version 9.4).

Procedures

BM219 was developed by Fudan University (Shanghai, China) and Biomissile (Anji) Pharmaceuticals Co., Ltd. (Anji, Zhejiang, China). BM219 is a freeze-dried formulation for nebulisation, and stored at a temperature of 2–8 °C prior to reconstitution with sterile water for use (3 ml for 60 mg; 6 ml for 120 mg). The placebo was composed of the BM219 formulation without antibody.

Participants entered the study with a previous positive SARS-CoV-2 viral test result. The investigator initially reviewed COVID-19-related symptoms, risk factors, and non-invasive inclusion and exclusion criteria. If the participant was eligible after this initial review, then staff at the site performed the invasive procedures, including blood samples, nasopharyngeal swab, and vital signs, to confirm eligibility. Enrolled participants were randomly assigned to placebo or BM219 on study day 1. The study consisted of a screening period of up to 5 days, a 5-day treatment period, and a subsequent 23-day observation period. Twice dose of 3 ml of either BM219 (60 mg) or placebo was administered to participants in cohort 1, one dose of 6 ml (120 mg) in cohort 2, twice dose of 6 ml (120 mg) in cohort 3. All participants received their assigned treatment via the Air360 mini + E nebulizer (Feellife, Shenzhen, China). The inhalation rate ranged from 0.21 to 0.43 ml/min, over approximately 7–13 min for 60 mg and 15–28 min for 120 mg BM219 or matching placebo. Nasopharyngeal swabs were collected by study staff, and COVID-19-related symptoms and clinical status were assessed by investigators at each site. Pharmacokinetic samples were collected on days 1–6. Induced sputum samples were collected on days 1 and 5. Safety blood laboratory tests (haematology, urinalysis, blood biochemistry, and coagulation) were also conducted.

Outcomes

The primary endpoint was the change in SARS-CoV-2 viral load from baseline to day 5, as measured by RT-PCR testing of nasopharyngeal swab samples. The secondary efficacy endpoints included: 1) proportion of the participants with viral load below the detection limit; 2) time from the first dose to the first negative nucleic acid test; 3) time from symptom onset to the first negative nucleic acid test; 4) time from the first dose to sustained clinical recovery (defined as the alleviation of all COVID-19–related symptoms to a total score of 0 or 1 for the sum of each symptom (on a scale from 0 to 3, with higher scores indicating greater severity; total scores on the 11-item scale range from 0 to 33) for 2 consecutive days; Supplementary Table S5); 5) progression from mild-to-moderate to severe or critical COVID-19, or patient death, within 28 days. All other endpoints are listed in the trial protocol.

Quantitative virology assay

Nasopharyngeal swabs were collected from all participants on day 1 before the first dose (i.e., baseline) and on days 2-5 to quantify the SARS-CoV-2 viral load through RT-PCR at the Teddy Clinical Research Laboratory (Jiangsu, China). The samples on day 1 were also used to sequence the viral genome using next-generation sequencing. SARS-CoV-2 nucleic acid test was conducted at screening, on day 1 before the first dose, days 2-5, 7, 10, 12, 14, and 18 to determine the Ct value. Conversion of Ct value to viral load (copies/ml) was performed using a method adapted from an IVD kit by Sensure Biotechnologies (China NMPA registration certificate number: 20203400064).

Pseudovirus neutralization assay

To evaluate neutralizing activity of BM219 against the JN.1 variant, the gene encoding spike (S) protein was commercially obtained and cloned into the pcDNA3.1 vector. Pseudoviruses were subsequently generated by co-transfecting 293T cells with spike-expressing plasmid and a luciferase-reporter HIV-1 backbone (pNL4-3.luc.RE). Supernatants were harvested after 48 h and filtered for use. For the neutralization assay, Huh-7 cells were infected with pseudoviruses pre-incubated with serially diluted BM219 antibody. Following 48 hours of incubation, luciferase activity was measured

to quantify infection. Neutralization was calculated by comparing relative light units (RLUs) of sample wells to virus and cell controls. The 50% inhibitory concentration (IC_{50}) was determined using nonlinear regression in GraphPad Prism.

Measurement of BM219 in serum and induced sputum

The concentration of BM219 antibody in serum and induced sputum from participants in both phases was validated using a sandwich-based enzyme-linked immunosorbent assay (ELISA). Specifically, recombinant SARS-CoV-2 sublineage BA.2 (Omicron) Spike RBD Protein (SinoBiological, Beijing, China) was coated onto an ELISA plate. BM219 standards and samples were added to the coated ELISA plate and incubated, during which BM219 was captured and immobilized on the plate. Subsequently, rabbit anti-BM219 polyclonal antibody (GenScript, Nanjing, China) was added to bind to BM219, followed by peroxidase-conjugated goat anti-rabbit IgG $F(ab')_2$ antibody to enable detection. After incubation, TMB substrate was added to initiate color development, and the reaction was terminated with 1 M sulfuric acid. Absorbance was measured at 450 nm for detection and 630 nm for reference using a Molecular Devices SpectraMax M5 plate reader. The quantification range of the assay was 0.50–16.00 ng/ml, with a lower limit of quantification (LLOQ) of 0.5 ng/ml.

Statistical analyses

Sample size was based on clinical and practical considerations rather than a formal statistical power calculation. The primary endpoint was analyzed using an analysis-of-covariance (ANCOVA) model, with baseline viral load as the covariate. Confidence intervals (CI) in this report were not adjusted for multiplicity. The Kaplan-Meier method was used to estimate the median time to sustained clinical recovery and negative nucleic acid test, and the 95% CI was estimated using the Brookmeyer-Crowley method with log–log transformation. Categorical variables were analyzed using $\chi 2$ test and Fisher's exact test. Statistical significance was set at $p<0.05$. All statistical analyses were performed using SAS software, version 9.4 (SAS Institute) and Phoenix WinNonlin version 8.3.

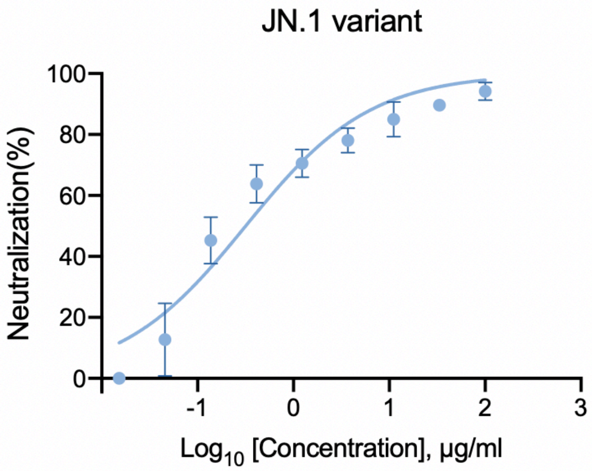

Variant	IC_{50} value (μg/ml)
JN.1	0.32
BA.2	0.69
BA.4	0.36
BA.5	1.50
BF.7	0.10
BQ.1.1	0.59
XBB	0.74

Figure S1. Neutralizing potency of BM219 against pseudoviruses of JN.1 variant. Pseudotyped viruses bearing JN.1 spike protein were preincubated with antibodies at the indicated concentrations and used to infect Huh-7 cells. Neutralization (%) was determined based on luciferase activities. The IC_{50} value was calculated using a nonlinear regression curve fit. Three independent experiments were performed in triplicate. Data indicate mean ± s.d.

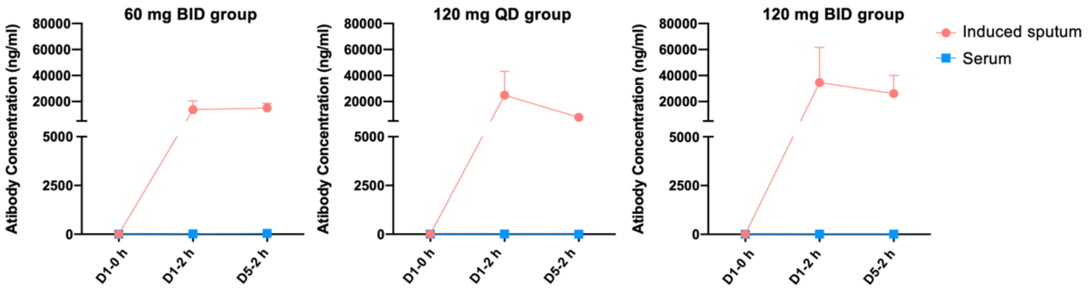

Figure S2. Antibody concentration in induced sputum and serum from BM219-treatment groups. Induced sputum and serum samples were collected from participants in the 60 mg BID, 120 mg QD, and 120 mg BID treatment groups at pre-inhalation (D1-0 h), 2 hours post-inhalation on Day 1 (D1-2 h), and 2 hours post-inhalation on Day 5 (D5-2 h). Antibody levels were quantified using an ELISA-based assay. Data are presented as mean ± s.d. based on three replicates per time point.

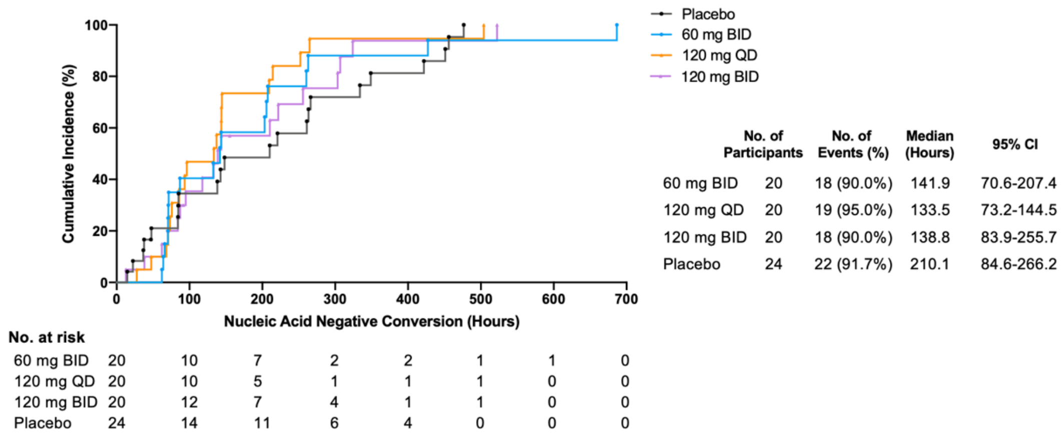

Figure S3. Kaplan–Meier curve for time to nucleic acid negative conversion in the full analysis population. Shown are the cumulative incidence of each endpoint over time (hours) for placebo, 60 mg BID, 120 mg QD, and 120 mg BID groups. Nucleic acid negative conversion represents the time from first dose to first negative RT-PCR test result. Data include number of participants at risk at each timepoint, event rates (n, %), median times with 95% confidence intervals, and comparative treatment trajectories.

Table S1. Number of patients enrolled in each site.

Center	No. of patients
The Third Hospital of Changsha	30
Hainan General Hospital	31
The Third Hospital of Hebei Medical University	18
The Third People's Hospital of Shenzhen and The Second Affiliated Hospital of Southern University of Science and Technology	5

Table S2. Demographic and baseline characteristics of patients.

Characteristic	60 mg BM219 (BID, N=20)	120 mg BM219 (QD, N=20)	120 mg BM219 (BID, N=20)	Placebo (N=24)	Total (N=84)
Age (mean ± s.d.)	32.5 ± 9.0	33.9 ± 9.6	32.1 ± 6.5	34.8 ± 9.6	33.4 ± 8.7
Media (IQR)	33.0	31.5	33.0	34.5	33.0
18-40	18 (90.0%)	17 (85.0%)	19 (95.0%)	20 (83.3%)	74 (88.1%)
41-64	2 (10.0%)	3 (15.0%)	1 (5.0%)	4 (16.7%)	10 (11.9%)
Sex					
Male	11 (55.0%)	11 (55.0%)	13 (65.0%)	10 (41.7%)	45 (53.6%)
Female	9 (45.0%)	9 (45.0%)	7 (35.0%)	14 (58.3%)	39 (46.4%)
BMI [a] (mean ± s.d.)	23.0 ± 2.4	22.4 ± 4.0	23.0 ± 3.9	22.1 ± 2.9	22.6 ± 3.3
Ct value of N gene (mean ± s.d.)	24.6 ± 4.6	23.7 ± 5.9	23.8 ± 4.8	22.5 ± 5.8	23.6 ± 5.3
Ct value of ORF gene (mean ± s.d.)	25.2 ± 4.7	24.4 ± 6.0	25.0 ± 4.8	23.2 ± 5.7	24.4 ± 5.3
SARS-CoV-2 infection					
JN.1	10 (50.0%)	13 (65.0%)	13 (65.0%)	14 (58.3%)	50 (59.5%)
EG.5	3 (15.0%)	0 (0)	1 (5.0%)	0 (0)	4 (4.8%)
Wild-type or other variants	2 (10.0%)	6 (30.0%)	5 (25.0%)	7 (29.2%)	20 (23.8%)
Unknown [b]	5 (25.0%)	1 (5.0%)	1 (5.0%)	3 (12.5%)	10 (11.9%)
High-risk factors					
Age >60 years	0 (0)	0 (0)	0 (0)	0 (0)	0 (0)
Pre-existing comorbidities [c]	0 (0)	2 (10.0%)	0 (0)	1 (4.2%)	3 (3.6%)
Immunocompromise	0 (0)	0 (0)	0 (0)	0 (0)	0 (0)
Obesity [d]	0 (0)	0 (0)	1 (5.0%)	0 (0)	1 (1.2%)
Smoking	0 (0)	0 (0)	0 (0)	0 (0)	0 (0)
COVID-19 vaccination status					
1 dose	5 (25.0%)	5 (25.0%)	5 (25.0%)	4 (16.7%)	19 (22.6%)
2 dose	3 (15.0%)	4 (20.0%)	4 (20.0%)	1 (4.2%)	12 (14.3%)
≥3 dose	9 (45.0%)	10 (50.0%)	10 (50.0%)	17 (70.1%)	46 (54.8%)
COVID-19 infection history	20 (100%)	20 (100%)	20 (100%)	24 (100%)	84 (100%)

[a] BMI denotes body-mass index.
[b] An unknown result indicates that the viral genome could not be identified.
[c] Comorbidities include cardiovascular disease (including hypertension), chronic lung disease, chronic kidney disease, active cancer, chronic kidney disease, and diabetes mellitus.
[d] Obesity is defined as a body-mass index of greater than 30.

Table S3. Adverse Events by System Organ Class (SOC).

SOC Category	60 mg BM219 (BID, N=20) n (%)	120 mg BM219 (QD, N=20) n (%)	120 mg BM219 (BID, N=20) n (%)	Placebo (N=24) n (%)	Total (N=84) n (%)
Total	2 (10.0)	9 (45.0)	7 (35.0)	10 (41.7)	23 (27.4)
General Disorders					
Triglycerides increased	0 (0)	1 (5.0)	0 (0)	3 (12.5)	4 (4.8)
Uric acid increased	0 (0)	0 (0)	1 (5.0)	2 (8.3)	3 (3.6)
High density lipoprotein (HDL) decreased	0 (0)	1 (5.0)	0 (0)	1 (4.2)	2 (2.4)
Neutrophils decreased	0 (0)	0 (0)	0 (0)	2 (8.3)	2 (2.4)
Bilirubin increased	0 (0)	0 (0)	1 (5.0)	0 (0)	1 (1.2)
Blood cholesterol increased	0 (0)	0 (0)	0 (0)	1 (4.2)	1 (1.2)
Platelets decreased	0 (0)	1 (5.0)	0 (0)	0 (0)	1 (1.2)
Bile acid increased	0 (0)	1 (5.0)	0 (0)	0 (0)	1 (1.2)
White blood cell (WBC) increased	0 (0)	0 (0)	0 (0)	1 (4.2)	1 (1.2)
Heart rate increased	0 (0)	1 (5.0)	0 (0)	0 (0)	1 (1.2)
Urine cast	0 (0)	0 (0)	1 (5.0)	0 (0)	1 (1.2)
Aspartate aminotransferase (AST) increased	0 (0)	0 (0)	0 (0)	1 (4.2)	1 (1.2)
Alanine aminotransferase (ALT) increased	0 (0)	0 (0)	0 (0)	1 (4.2)	1 (1.2)
Metabolic and Nutritional Disorders					
Hyperlipidemia	1 (5.0)	2 (10.0)	0 (0)	0 (0)	3 (3.6)
Hypertriglyceridemia	0 (0)	0 (0)	2 (10.0)	0 (0)	2 (2.4)
Hyperuricemia	1 (5.0)	1 (5.0)	0 (0)	0 (0)	2 (2.4)
Blood and Lymphatic System Disorders					
Anaemia	1 (5.0)	1 (5.0)	0 (0)	1 (4.2)	3 (3.6)
Gastrointestinal Disorders					
Abdominal Distension	0 (0)	0 (0)	0 (0)	1 (4.2)	1 (1.2)
Dyspepsia	0 (0)	0 (0)	0 (0)	1 (4.2)	1 (1.2)
Eye Disorders					
Conjunctivitis	0 (0)	0 (0)	1 (5.0)	0 (0)	1 (1.2)

Table S4. Key virologic end points in full analysis set population.

	60 mg BM219 (BID, N=20)	Matching Placebo (N=8)	120 mg BM219 (QD, N=20)	Matching Placebo (N=8)	120 mg BM219 (BID, N=20)	Matching Placebo (N=8)
The average change in viral load from baseline to day 5						
Least-squares mean change (log_{10} copies/ml)	-3.12	-3.05	-3.46	-3.16	-2.70	-3.01
95% CI	-3.94 to -2.31	-4.34 to -1.75	-4.10 to -2.81	-4.16 to -2.16	-3.66 to -1.73	-4.65 to -1.38
Difference vs. placebo						
Least-squares mean (log_{10} copies/ml)	-0.08	/	-0.29	/	0.32	/
95% CI	-1.61 to 1.46	/	-1.49 to 0.9	/	-1.59 to 2.22	/

Table S5. COVID-19 Related Symptoms.

Items	Scoring*
1. Fever	None = 0<br>Mild = 1<br>Moderate = 2<br>Severe = 3
2. Cough	
3. Sore throat	
4. Stuffy or runny nose	
5. Headache	
6. Muscle or body aches	
7. Shortness of breath (difficulty breathing)	
8. Nausea (feeling like to throw up)	
9. Chills or shivering	
10. Low energy or tiredness	
11. Diarrhea	

* Score values are included in the table for ease of reference. A higher score indicates worse symptoms[1].

Supplementary References

1. FDA CDER, CBER. Assessing COVID-19-Related Symptoms in Outpatient Adult and Adolescent Subjects in Clinical Trials of Drugs and Biological Products for COVID 19 Prevention or Treatment Guidance for Industry. Sept, 2020. Accessed on August 25, 2022 from https://www.fda.gov/media/142143/download

Phase 2 Study Protocol

**Protocol No.**	BM219-201
**Registration Category**	Category 1 Therapeutic Biological Products
**Sponsor**	Shanghai Biomissile Biotech Co., Ltd.
**Study Title**	A Randomised, Double-blind, Placebo-controlled, Placebo Parallel-Controlled, Multicenter Phase II Clinical Study to Evaluate the Safety and Efficacy of BM219 in Patients with Mild and Moderate COVID-19
**Study Phase**	Phase II
**Study Drug**	BM219, lyophilized formulation (for nebulization)<br>Placebo
**Study Objectives**	**Primary Objective**<br>To preliminarily evaluate the efficacy of BM219 administered via inhalation, and explore its optimal dosage, using placebo as a control.<br>**Secondary Objectives**<br>(1) To evaluate the pharmacokinetic (PK) characteristics of multiple nebulized inhalations of BM219 in patients with mild and moderate coronavirus disease 2019 (COVID-19).<br>(2) To evaluate the immunogenicity of multiple nebulized inhalations of BM219 in patients with mild and moderate COVID-19.<br>(3) To evaluate the safety of multiple nebulized inhalations of BM219 in patients with mild and moderate COVID-19.
**Evaluation Endpoints**	**Primary Efficacy Endpoint:**<br>(1) To evaluate the mean change from baseline in viral load in nasopharyngeal swabs within 5 days after treatment;<br>**Secondary Efficacy Endpoints:**<br>(2) Proportion of subjects with viral load below the limit of detection at each time point;<br>(3) Time from first dose to first negative nucleic acid test;<br>(4) Time from initial symptom onset to first negative nucleic acid test;<br>(5) Time to sustained clinical recovery;<br>(6) Proportion of subjects with mild and moderate COVID-19 who have progressed to severe or critical illness, or mortality rate;<br>**Other Measures:**<br>(1) PK parameters of BM219 in patients with mild and moderate COVID-19.<br>(2) Immunogenicity of BM219 in patients with mild and

	moderate COVID-19: analyze the production time and titers of anti-drug antibodies (ADAs); ADA-positive samples need to be tested for neutralising antibodies (Nabs).<br>(3) The safety of BM219 in patients with mild and moderate COVID-19, including adverse events (AEs) and serious adverse events (SAEs), local irritation reactions, vital signs, physical examination, laboratory tests (hematology, blood chemistry, coagulation function, etc.), and 12-lead electrocardiogram (ECG), etc.<br>(4) Changes in BM219 concentration in induced sputum over time.
**Overall Design**	This study plans to enroll 84 patients with mild and moderate COVID-19.<br>This is a randomised, double-blind, placebo-controlled, multicenter clinical study to evaluate the efficacy and safety of BM219 in patients with mild and moderate COVID-19, and to explore its optimal dosage. Enrolled subjects must have tested positive for COVID-19 nucleic acid. A total of 84 subjects with mild and moderate COVID-19 are planned to be enrolled, including 20 subjects each in the investigational groups of 60 mg BID, 120 mg QD, and 120 mg BID, and 24 subjects in the placebo group. Patients in all dose groups will be enrolled in parallel.<br>Subjects will undergo screening from Day -5 to Day -1 before dosing. Subjects will undergo pre-dose assessments during the screening period. If subjects meet the criteria for participation in this clinical study, they will be randomised on Day 1 (or Day -1) and receive BM219 or placebo.<br>Subjects will be hospitalized as specified by the study site or return to the clinical site at specified time points for appropriate visits. Subjects will be followed up until 28 days after the first dose and should complete the visits specified in the protocol. After the completion of the visits, the subjects will withdraw from the study.
**Route of Administration**	Nebulized inhalation
**Dosing Cycle**	Each subject will be administered 5 or 10 doses. Each subject will receive the corresponding dosage based on his/her assigned dose group. The 120 mg (QD) group will be administered once daily (every 24h±4h) for 5 consecutive days, with a total of 5 doses; the 60 mg (BID) and 120 mg (BID) groups will be administered twice daily for 10 consecutive doses. For BID dosing groups, if the first dose is administered after 12:00 noon on the dosing day, it will be administered only once that day and from the second day onward twicely daily until the 10th dose (with an interval of 12h±2h).

**Study Period**	Screening period of 1-5 days, drug administration for 5 or 6 days, and then follow-up until 28 days after the first dose
**Sample Size**	84 patients with mild and moderate COVID-19
**Inclusion and Exclusion Criteria**	**Inclusion Criteria:**<br>(1) Patients aged ≥18 years and ≤70 years;<br>(2) Male or female patients;<br>(3) Patients with documented evidence of infection with novel coronavirus (SARS-CoV-2) at screening: infection confirmed by molecular testing (antigen or nucleic acid) of any respiratory specimen (such as nasal swab or saliva) at screening;<br>(4) Patients diagnosed with mild or moderate COVID-19 (antigen or nucleic acid positive);<br>(5) Patients with COVID-19 target symptoms ≤ 3 days prior to randomisation and at least one COVID-19 target symptom still present at the time of randomisation (with COVID-19 target symptoms including pyrexia, cough, nasal obstruction or rhinorrhoea, sore throat or dry throat, shortness of breath or dyspnoea, headache, muscle or generalized soreness, diarrhoea, chills, nausea, vomiting, etc.);<br>(6) Patients with Ct values ≤30 for the N and ORF genes in COVID-19 nucleic acid tests at screening;<br>(7) Patients who agree not to participate in other clinical trials during the study;<br>(8) Patients who commit to having no plans for conception, sperm or egg donation and voluntarily agree to use effective physical contraception measures (including their partners) during the study period and for 6 months after the last dose;<br>(9) Patients who are able to understand the study procedures, possible benefits and potential risks, and voluntarily participate in the study; are able to communicate well with the investigators, comply with the requirements of the entire study, and have signed the written informed consent form (ICF).<br>**Exclusion Criteria:**<br>(1) Patients who have received SARS-CoV-2 antiviral drugs within 14 days prior to screening, or who have received (within 30 days prior to randomization or within 5 drug half-lives, whichever is longer) or are expected to receive (during the study) SARS-CoV-2 monoclonal antibody or COVID-19 convalescent plasma;<br>(2) Patients with severe or critical COVID-19 who meet any of the following criteria:<br>a. Polypnoea, with respiratory rate (RR) ≥30 breaths/min;<br>b. At rest, oxygen saturation ≤ 93% when breathing air;

   c. Progressive aggravation of clinical symptoms, and significant progression of lesions by more than 50% within 24-48 hours as shown by pulmonary imaging;
   d. Respiratory failure requiring mechanical ventilation;
   e. Shock;
   f. Other concurrent organ failures, etc.

(3) Patients with suspected or confirmed acute systemic infection other than COVID-19 (e.g., concomitant influenza suggested by etiology test; high likelihood of bacterial infection based on symptoms, signs, laboratory tests, or imaging) that may interfere with the assessment of response to study intervention at screening;

(4) Patients with immunodeficiency (e.g., AIDS, long-term use of corticosteroids or other immunosuppressive drugs);

(5) Patients with a history of allergy to any antibody drug, or known allergy to components of the investigational product or placebo;

(6) Patients who have received a COVID-19 vaccine within 3 months prior to screening or plan to receive a COVID-19 vaccine during the study;

(7) Patients with a history of active pathological haemorrhage (e.g., peptic ulcer, intracranial haemorrhage, etc.), or those with haemorrhagic diathesis (e.g., coagulation disorders, recurrent gingival bleeding, etc.);

(8) Patients with laboratory tests that meet the following conditions at screening:
   a. Alanine aminotransferase (ALT), aspartate aminotransferase (AST), alkaline phosphatase (ALP), or gamma-glutamyltransferase (GGT) ≥2.5×ULN, and total bilirubin ≥1.5×ULN;
   b. Estimated glomerular filtration rate [eGFR] < 45 mL/min/1.73 m^2 (calculated using the [CKD-EPI] formula);
   c. Fasting blood glucose >11.1 mmol/L；
   d. Haemoglobin (Hb) < 8.0 g/dL;
   e. Any of the following: platelet count (PLT) < 50×10^9/L; absolute neutrophil count < 1.0×10^9/L.

(9) Patients who are initially screened positive for human immunodeficiency virus (HIV) and tested positive for treponema pallidum antibodies at screening, or those known to have active liver disease (e.g., acute or chronic hepatitis B, hepatitis C, PBC, hepatic cirrhosis, or acute liver failure, except for nonalcoholic fatty liver disease).

(10) Patients who have had major trauma or undergone major surgery within 3 months prior to screening, or have not recovered from surgery, or have undergone surgery that may

	significantly affect the in vivo process or safety evaluation of the study drug, or are expected to have surgeries planned during the study;<br>(11) Patients who have used other investigational drugs or devices within 90 days prior to screening, or who plan to participate in other drug or device clinical trials during this study;<br>(12) Pregnant or breastfeeding women or those with a positive pregnancy test;<br>(13) Patients with a history of needle sickness and blood sickness, or those who are unsuitable for venous blood collection;<br>(14) Patients with any other factors unsuitable for participation in this study as judged by the investigator, such as other serious diseases (including psychiatric disorders), serious laboratory abnormalities, family or social factors that may affect the subject's safety or blood sample collection.
**Withdrawal Criteria**	**(1) Withdrawal at the Investigator's Discretion**<br>During the study, an enrolled subject experience the following conditions, under which it is inappropriate to continue the study, and the investigator decides to withdraw the subject from the study.<br>1) The subject experiences an AE or SAE, which may have an impact on the study results or renders him/her inappropriate to continue in the study in the opinion of the investigator;<br>2) The subject has used other medications, which may have an impact on the study results or renders him/her inappropriate to continue in the study in the opinion of the investigator;<br>3) Poor subject compliance, including but not limited to:<br>a) The subject does not take medications or undergo examinations according to the protocol;<br>b) The subject does not take effective non-drug contraception measures during the study, which may have an impact on the study results or renders him/her inappropriate to continue in the study in the opinion of the investigator;<br>4) The subject has other behaviors affecting the efficacy, safety, and PK results of the study, which may have an impact on the study results or renders him/her inappropriate to continue in the study in the opinion of the investigator;<br>5) The subject has other conditions that, in the opinion of the investigator, withdrawal from the study is most beneficial to him/her or that it is inappropriate to continue the study.

	**(2) Withdrawal at the request of the subject**<br>According to the provisions of the ICF, the subject has the right to withdraw from the study during the study, or becomes lost to follow-up by no longer receiving medication and testing. Every effort should be made to understand the reasons for their withdrawal and record them.
**Study Termination Criteria**	(1) There occur significant unexpected AEs during the study, for which the investigators consider it necessary to terminate the study.<br>(2) There occur serious violations of the clinical study protocol, Good Clinical Practice (GCP), or relevant regulatory requirements during the study, for which the study needs to be terminated.<br>(3) The sponsor requests termination of the study (e.g., due to reasons such as funding, administration, efficacy, etc.) while sufficiently ensuring the benefits and safety of the subjects.<br>(4) The National Medical Products Administration or the Ethics Committee has ordered the termination of the study for certain reasons.
**Concomitant Medications and Therapies**	**Prohibited Therapies**<br>• From randomization to Day 28 of the study, subjects are prohibited from receiving SARS-CoV-2 antiviral treatments (such as Nirmatrelvir tablets/Ritonavir tablets, Molnupiravir capsules, Azvudine tablets, Simnotrelvir tablets/Ritonavir tablets, Deuremidevir Hydrobromide tablets, Leritrelvir tablets, etc.);<br>• From randomization to Day 28 of the study, the use of COVID-19 convalescent plasma or SARS-CoV-2 monoclonal antibodies is prohibited;<br>• From randomization until Day 28 of the study, the use of medications to alleviate COVID-19 symptoms is prohibited: antipyretic/analgesic drugs, antitussive drugs, nasal obstruction/rhinorrhoea, compound cold medications, antihistamines**, antibacterial and antifungal drugs (except for complications of suspected bacterial or fungal infections after Day 1 of treatment), glucocorticoids**, interleukin-6 (IL-6) inhibitors (including tocilizumab), immunosuppressants, Chinese herbal medicines/patent medicines that have an auxiliary effect in alleviating COVID-19 symptoms, except for medications permitted in the 'Guidelines for Symptomatic Relief Medication during the Study' (see Appendix 2);<br>Note: ** The use of topical preparations is permitted, but use in the eyes, nose, ears, or via inhalation is prohibited.<br>• From randomization to Day 28 of the study, it is prohibited to receive TCM treatments (e.g., acupuncture) or TCM physiotherapy (e.g., cupping, etc.) that alleviate COVID-19

	symptoms;<br>• From randomization to Day 28 of the study, the use of other investigational drugs is prohibited.<br>**Permitted Therapies**<br>Subjects may receive treatments permitted in the "Diagnosis and Treatment Protocol for Novel Coronavirus Pneumonia (Trial Version 10)", except for contraindicated drugs or drugs with potential drug-drug interactions. If a subject progresses to severe/critical COVID-19 during the study, treatment according to local guidelines for severe/critical COVID-19 is allowed, and investigators are advised to contact the sponsor or CRO medical monitor for discussion.<br>**Symptomatic Treatment**<br>The recovery of COVID-19 related symptoms is a secondary endpoint of this study; if the subject's related symptoms are mild, interventions will not be given unless necessary, and it is recommended to observe for 1~2 days first; if the subject still cannot tolerate and strongly requests medication, the drug can be considered for intervention as specified in Appendix 2, but should be avoided as far as possible.<br>If symptomatic medications are used, the dosage, date, and time of each administration should be recorded. Measurement of body temperature and evaluation of COVID-19 symptoms will be conducted before or more than 4 hours after symptomatic treatment.
**Statistical Analysis**	This is an exploratory study. All study data will be presented in the form of data listings. Statistical analyses will primarily be descriptive. Descriptive statistics will be used for continuous variables, while frequencies and percentages will be used for categorical and ordinal variables. Percentages will be based on the number of non-missing values within each dose group.

Clinical trial register information

BM219 Phase II clinical trial (BM219-101) in www.chinadrugtrials.org.cn

Version V1.0 Date: 2023.07.10

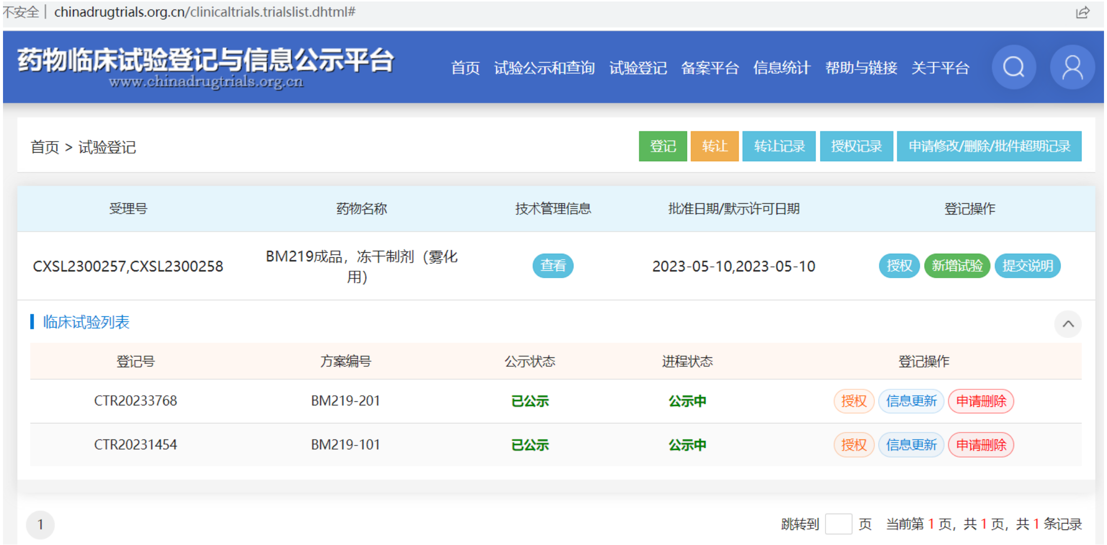

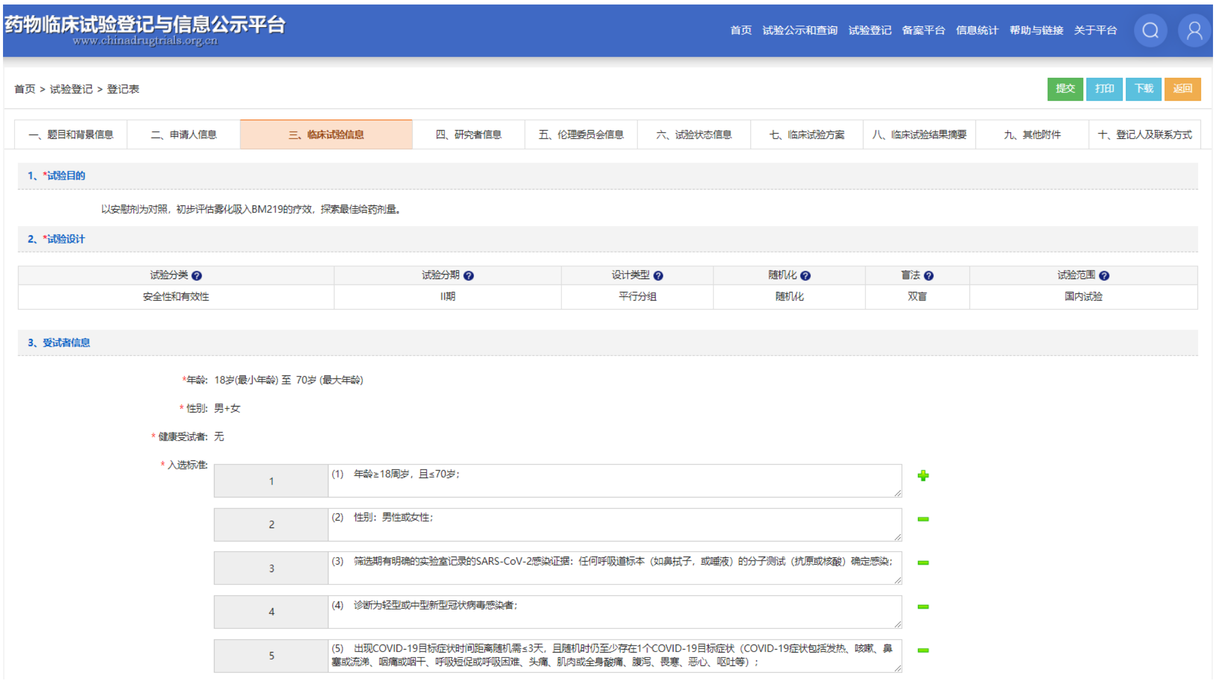

1. Objectives of Study					
To preliminarily evaluate the efficacy of BM219 administered via nebulized inhalation, and explore its optimal dosage, using placebo as a control.					
2.Study design					
Classification of study	Study phase	Study design	Randomization	Blinding	Study range
Safety and efficacy	Phase II	Parallel	Randomization	Double-blind	Domestic test
3. Information of the subjects					
age：From 18 years old (the minimum age) to 70 years old (the maximum age)					
Gender：Both male and female					

Healthy subjects: None		
Inclusion criteria:	1	(1) Patients aged ≥18 years and ≤70 years;
	2	(2) Male or female patients;
	3	(3) Patients with documented evidence of infection with novel coronavirus (SARS-CoV-2) at screening: infection confirmed by molecular testing (antigen or nucleic acid) of any respiratory specimen (such as nasal swab or saliva) at screening;
	4	(4) Patients diagnosed with mild or moderate COVID-19;
	5	(5) Patients with COVID-19 target symptoms ≤ 3 days prior to randomization and at least one COVID-19 target symptom still present at the time of randomization (with COVID-19 target symptoms including pyrexia, cough, nasal obstruction or rhinorrhoea, sore throat or dry throat, shortness of breath or dyspnoea, headache, muscle or generalized soreness, diarrhoea, chills, nausea, vomiting, etc.);

	6	(6) 筛选期受试者新型冠状病毒核酸检测N基因和ORF基因Ct值<25；
	7	(7) 受试者同意在研究期间不参加其他临床试验；
	8	(8) 受试者承诺在试验期间及末次用药后6个月内无生育、捐献精子或卵子计划且自愿采取有效物理避孕措施（包括伴侣）；
	9	(9) 能够理解研究程序、可能的受益和潜在的风险，并自愿参与研究；能与研究者良好沟通，遵从整个研究的要求，且签署了书面的知情同意书。
* 排除标准:	1	(1) 筛选前14天内使用过SARS-CoV-2抗病毒药物；或已接受（随机前30天内或5个药物半衰期内，以较长者为准）或研究期间预期接受SARS-CoV-2单克隆抗体或恢复期COVID-19患者血浆治疗；
	2	(2) 重型或危重型新型冠状病毒感染者，符合以下任意一条：<br>a. 出现气促，RR≥30次/分；<br>b. 静息状态下，呼吸空气时，血氧饱和度≤93%；<br>c. 临床症状进行性加重，肺部影像学显示24-48小时内病灶明显进展＞50%者；<br>d. 出现呼吸衰竭，且需要机械通气；<br>e. 出现休克；<br>f. 合并其他器官功能衰竭等。
	3	(3) 筛选时有除COVID-19以外的疑似或确诊的急性全身性感染（例如病原学检查提示合并流感；根据症状、体征、实验室检查或影像学提示有细菌感染的高度可能性），可能会干扰对研究干预反应的评估；
	4	(4) 免疫功能缺陷（如艾滋病患者，长期使用皮质类固醇或其他免疫抑制药物）；
	5	(5) 以前对任何抗体药物过敏，或已知对试验药物或安慰剂的成分过敏；
	6	(6) 筛选前3个月内接种过新冠疫苗或者计划在试验期间接种新冠疫苗的患者；
	7	(7) 活动性病理性出血史者（如消化性溃疡、颅内出血等），或有出血倾向者（如凝血功能障碍、反复牙龈出血等）；

Inclusion criteria:	6	(6) Patients with Ct values < 25 for the N and ORF genes in COVID-19 nucleic acid tests at screening;
	7	(7) Patients who agree not to participate in other clinical trials during the study;
	8	(8) Patients who commit to having no plans for conception, sperm or egg donation and voluntarily agree to use effective physical contraception measures (including their partners) during the study period and for 6 months after the last dose;
	9	(9) Patients who are able to understand the study procedures, possible benefits and potential risks, and voluntarily participate in the study; are able to communicate well with the investigators, comply with the requirements of the entire study, and have signed the written informed consent form (ICF).
Exclusion criteria:	1	(1) Patients who have received SARS-CoV-2 antiviral drugs within 14 days prior to screening, or who have received (within 30 days prior to randomization or within 5 drug half-lives, whichever is longer) or are expected to receive (during the study) SARS-CoV-2 monoclonal antibody or COVID-19 convalescent plasma;

	2	(2) Patients with severe or critical COVID-19 who meet any of the following criteria:<br>a. Polypnoea, with respiratory rate (RR) ≥30 breaths/min;<br>b. At rest, oxygen saturation ≤ 93% when breathing air;<br>c. Progressive aggravation of clinical symptoms, and significant progression of lesions by more than 50% within 24-48 hours as shown by pulmonary imaging;<br>d. Respiratory failure requiring mechanical ventilation;<br>e. Shock;<br>f. Other concurrent organ failures, etc.
	3	(3) Patients with suspected or confirmed acute systemic infection other than COVID-19 (e.g., concomitant influenza suggested by etiology test; high likelihood of bacterial infection based on symptoms, signs, laboratory tests, or imaging) that may interfere with the assessment of response to study intervention at screening;
	4	(4) Patients with immunodeficiency (e.g., AIDS, long-term use of corticosteroids or other immunosuppressive drugs);
	5	(5) Patients with a history of allergy to any antibody drug, or known allergy to components of the investigational product or placebo;
	6	(6) Patients who have received a COVID-19 vaccine within 3 months prior to screening or plan to receive a COVID-19 vaccine during the study;
	7	(7) Patients with a history of active pathological haemorrhage (e.g., peptic ulcer, intracranial haemorrhage, etc.), or those with haemorrhagic diathesis (e.g., coagulation disorders, recurrent gingival bleeding, etc.);

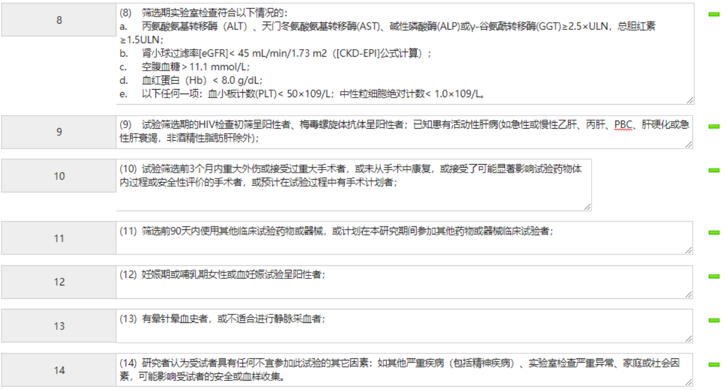

8	(8) 筛选期实验室检查符合以下情况的:<br>a. 丙氨酸氨基转移酶（ALT）、天门冬氨酸氨基转移酶(AST)、碱性磷酸酶(ALP)或γ-谷氨酰转移酶(GGT)≥2.5×ULN，总胆红素≥1.5ULN;<br>b. 肾小球过滤率[eGFR]< 45 mL/min/1.73 m2（[CKD-EPI]公式计算）；<br>c. 空腹血糖＞11.1 mmol/L;<br>d. 血红蛋白（Hb）< 8.0 g/dL;<br>e. 以下任何一项：血小板计数(PLT)< 50×109/L；中性粒细胞绝对计数< 1.0×109/L。
9	(9) 试验筛选期的HIV检查初筛呈阳性者、梅毒螺旋体抗体呈阳性者；已知患有活动性肝病(如急性或慢性乙肝、丙肝、PBC、肝硬化或急性肝衰竭，非酒精性脂肪肝除外)；
10	(10) 试验筛选前3个月内重大外伤或接受过重大手术者，或未从手术中康复，或接受了可能显著影响试验药物体内过程或安全性评价的手术者，或预计在试验过程中有手术计划者；
11	(11) 筛选前90天内使用其他临床试验药物或器械，或计划在本研究期间参加其他药物或器械临床试验者；
12	(12) 妊娠期或哺乳期女性或血妊娠试验呈阳性者；
13	(13) 有晕针晕血史者，或不适合进行静脉采血者；
14	(14) 研究者认为受试者具有任何不宜参加此试验的其它因素：如其他严重疾病（包括精神疾病）、实验室检查严重异常、家庭或社会因素，可能影响受试者的安全或血样收集。

Exclusion criteria：	8	(8) Patients with laboratory tests that meet the following conditions at screening:<br>a. Alanine aminotransferase (ALT), aspartate aminotransferase (AST), alkaline phosphatase (ALP), or gamma-glutamyltransferase (GGT) ≥2.5×ULN, and total bilirubin ≥1.5×ULN;<br>b. Estimated glomerular filtration rate [eGFR] < 45 mL/min/1.73 m^2 (calculated using the [CKD-EPI] formula);<br>c. Fasting blood glucose >11.1 mmol/L;<br>d. Haemoglobin (Hb) < 8.0 g/dL;

		e. Any of the following: platelet count (PLT) < 50×10^9/L; absolute neutrophil count < 1.0×10^9/L.
	9	(9) Patients who are initially screened positive for human immunodeficiency virus (HIV) and tested positive for treponema pallidum antibodies at screening, or those known to have active liver disease (e.g., acute or chronic hepatitis B, hepatitis C, PBC, hepatic cirrhosis, or acute liver failure, except for nonalcoholic fatty liver disease).
	10	(10) Patients who have had major trauma or undergone major surgery within 3 months prior to screening, or have not recovered from surgery, or have undergone surgery that may significantly affect the in vivo process or safety evaluation of the study drug, or are expected to have surgeries planned during the study;
	11	(11) Patients who have used other investigational drugs or devices within 90 days prior to screening, or who plan to participate in other drug or device clinical trials during this study;
	12	(12) Pregnant or breastfeeding women or those with a positive pregnancy test;
	13	(13) Patients with a history of needle sickness and blood sickness, or those who are unsuitable for venous blood collection;
	14	(14) Patients with any other factors unsuitable for participation in this study as judged by the investigator, such as other serious diseases (including psychiatric disorders), serious laboratory abnormalities, family or social factors that may affect the subject's safety or blood sample collection.

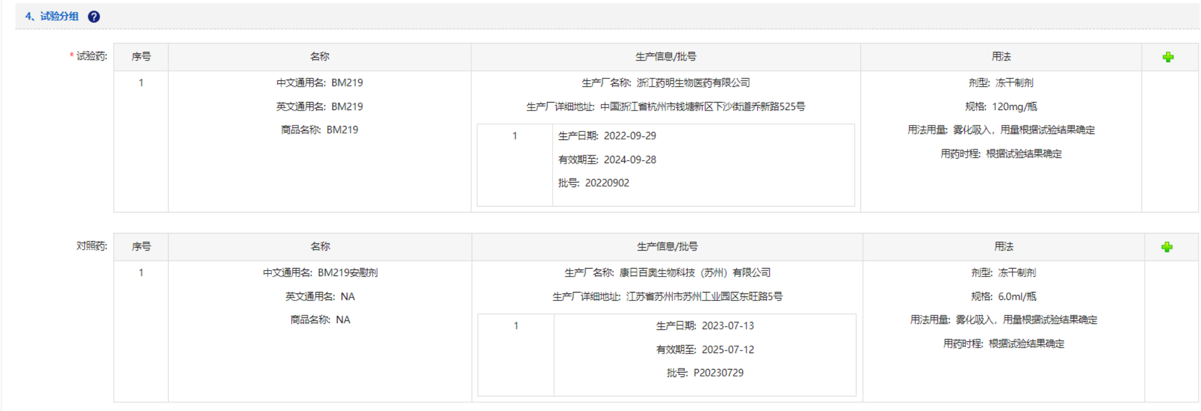

4、试验分组

* 试验药:

序号	名称	生产信息/批号	用法
1	中文通用名: BM219<br>英文通用名: BM219<br>商品名称: BM219	生产厂名称: 浙江药明生物医药有限公司<br>生产厂详细地址: 中国浙江省杭州市钱塘新区下沙街道乔新路525号<br>1 生产日期: 2022-09-29<br>有效期至: 2024-09-28<br>批号: 20220902	剂型: 冻干制剂<br>规格: 120mg/瓶<br>用法用量: 雾化吸入，用量根据试验结果确定<br>用药时程: 根据试验结果确定

对照药:

序号	名称	生产信息/批号	用法
1	中文通用名: BM219安慰剂<br>英文通用名: NA<br>商品名称: NA	生产厂名称: 康日百奥生物科技（苏州）有限公司<br>生产厂详细地址: 江苏省苏州市苏州工业园区东旺路5号<br>1 生产日期: 2023-07-13<br>有效期至: 2025-07-12<br>批号: P20230729	剂型: 冻干制剂<br>规格: 6.0ml/瓶<br>用法用量: 雾化吸入，用量根据试验结果确定<br>用药时程: 根据试验结果确定

4. Trial grouping

investigational drug:

serial number	name	Production Information/Batch Number	usage
1	Chinese Generic Name: BM219	Manufacture : WuXi Biologics (Zhejiang) Pharmaceutical Co., Ltd. Address: No. 525, QiaoXin Rd.,	Dosage form: lyophilized powder Specifications:

	English Generic Name: BM219 Trade Name: BM219	Xiasha Street, Hangzhou, Zhejiang, China<br>1 Date of Manufacture:2022-09-29<br>Valid until:2024-09-28<br>Batch No.: 20220902	120mg/Vial<br>Usage and Dosage: Nebulized inhalation. The dosage will be determined according to the test results.<br>Treatment Duration: To be determined according to the test results.

control drug:

serial number	name	Production Information/Batch Number	usage
1	Chinese Generic Name: BM219 Placebo English Generic Name: NA Trade Name: NA	Manufacture : Bioworkshops (Suzhou) Limited<br>Address: No. 5 Dongwang Road, Suzhou Industrial Park, Suzhou, Jiangsu<br>1 Date of Manufacture:2023-07-13<br>Valid until:2025-07-12<br>Batch No.: P20230729	Dosage form: lyophilized powder<br>Specifications: 6.0ml/Vial<br>Usage and Dosage: Nebulized inhalation. The dosage will be determined according to the test results.<br>Treatment Duration: To be determined according to the test results.

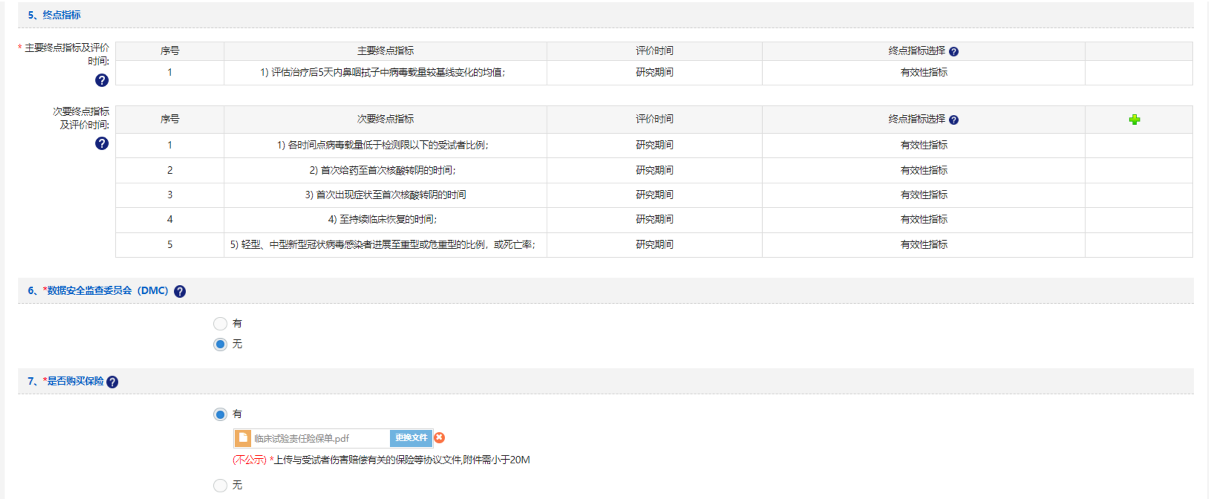

5、终点指标

*主要终点指标及评价时间:

序号	主要终点指标	评价时间	终点指标选择	
1	1) 评估治疗后5天内鼻咽拭子中病毒载量较基线变化的均值;	研究期间	有效性指标	

次要终点指标及评价时间:

序号	次要终点指标	评价时间	终点指标选择	
1	1) 各时间点病毒载量低于检测限以下的受试者比例;	研究期间	有效性指标	
2	2) 首次给药至首次核酸转阴的时间;	研究期间	有效性指标	
3	3) 首次出现症状至首次核酸转阴的时间	研究期间	有效性指标	
4	4) 至持续临床恢复的时间;	研究期间	有效性指标	
5	5) 轻型、中型新型冠状病毒感染者进展至重型或危重型的比例，或死亡率;	研究期间	有效性指标	

6、*数据安全监查委员会（DMC）

有
无

7、*是否购买保险

有
临床试验责任险保单.pdf 更换文件
(不公示) *上传与受试者伤害赔偿有关的保险等协议文件,附件需小于20M
无

5. Endpoints

Primary Endpoint Indicators and Evaluation Time:

serial number	primary endpoint indicators	evaluation time	type
1	1) To evaluate the mean change from baseline in viral load in nasopharyngeal swabs within 5 days after treatment	during the research period	efficacy indicator

Secondary Endpoint Indicators and Evaluation Time:

serial number	secondary endpoint indicators	evaluation time	type
1	1) Proportion of subjects with viral load below the limit of detection at each time point;	during the research period	efficacy indicator

2	2) Time from first administration to first negative nucleic acid test;	during the research period	efficacy indicator
3	3) Time from initial symptom onset to first negative nucleic acid test;	during the research period	efficacy indicator
4	4) Time to sustained clinical recovery;	during the research period	efficacy indicator
5	5) Proportion of subjects with mild and moderate COVID-19 who have progressed to severe or critical illness, or mortality rate	during the research period	efficacy indicator

6. Data Monitoring Committee（DMC）
No
7. Whether to purchase insurance
Yes
